# Supplementary figures and images for: MACF1 controls skeletal muscle function through the microtubule-dependent localization of extra-synaptic myonuclei and mitochondria biogenesis
Source: eLife. 2021 Aug 27;10:e70490. doi: 10.7554/eLife.70490 (PMC8500715; doi:10.7554/eLife.70490)

MACF1

GM

3

3

5

5

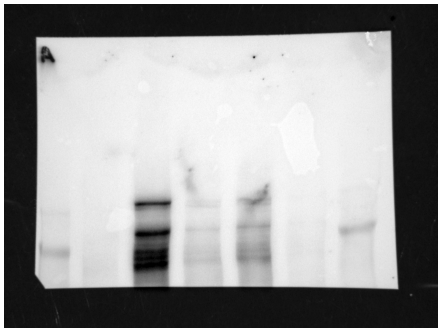

GAPDH

GM

3

3

5

5

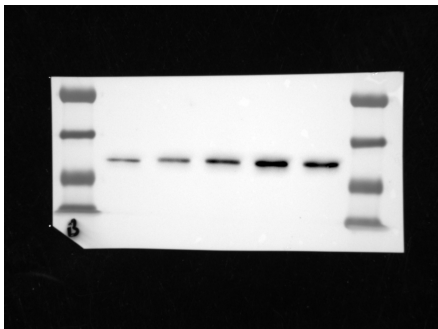

Supplement: Figure 1—source data 2. [file elife-70490-fig1-data2.pdf]

MACF1

GM 3 3 5 5

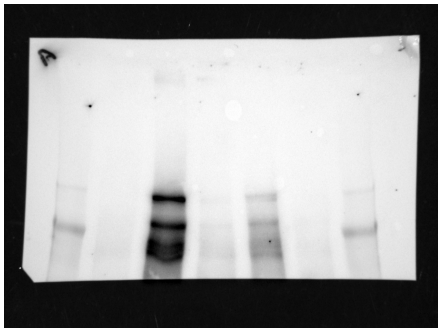

GAPDH

GM 3 3 5 5

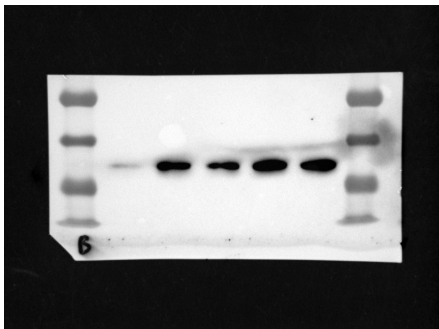

Supplement: Figure 1—figure supplement 1—source data 2. [file elife-70490-fig1-figsupp1-data2.pdf]
