# Supplementary material for: MACF1 controls skeletal muscle function through the microtubule-dependent localization of extra-synaptic myonuclei and mitochondria biogenesis
Source: eLife. 2021 Aug 27;10:e70490. doi: 10.7554/eLife.70490 (PMC8500715; doi:10.7554/eLife.70490)

OXPHOS

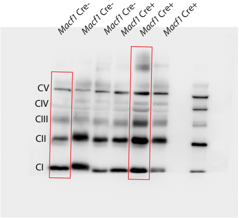

TOM20

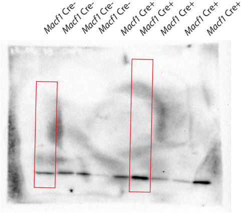

Actin

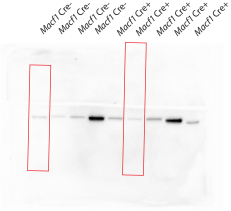

Supplement: Figure 7—source data 2. [file elife-70490-fig7-data2.pdf]
